# Oral administration of titanium dioxide nanoparticle through ovarian tissue alterations impairs mice embryonic development

**Published:** 2018-06

**Authors:** Mojtaba Karimipour, Masoumeh Zirak Javanmard, Abbas Ahmadi, Abbas Jafari

**Affiliations:** 1 *Department of Anatomy and Histology, Faculty of Medicine, Urmia University of Medical Sciences, Urmia, Iran.*; 2 *Department of Basic Sciences, Faculty of Veterinary Medicine, Urmia University, Urmia, Iran.*; 3 *Department of Occupational Health, School of Health, Urmia University of Medical Sciences, Urmia, Iran.*

**Keywords:** Nanoparticle, Titanium dioxide, Ovary, In vitro fertilization, Mice

## Abstract

**Background:**

Titanium dioxide nanoparticle (TiO_2_NP) is commonly used in industrial products including food colorant, cosmetics, and drugs. Previous studies have shown that oral administration of TiO_2_NP can be toxic to the reproductive system, but little is known if TiO_2_NP could be able to affect the functions of the female reproductive system, in particular fertility.

**Objective:**

The objective was to evaluate the effects of oral administration of TiO_2_NP on histological changes in ovaries, pregnancy rate and in vitro fertility in mice.

**Materials and Methods:**

In this experimental study, 54 adult female NMRI mice were randomly assigned to two groups: control group (received vehicle orally) and TiO_2_NP group (received 100 mg/kg/daily TiO_2_NP solution orally). After 5 wk, pregnancy and in vitro fertilization rates, histological changes in ovaries, malondyaldehyde and estrogen hormone levels in the blood serum were investigated and compared between groups.

**Results:**

Our results revealed that TiO_2_NP administration induced histological alterations in ovary including, degenerating and reduction of ovarian follicles, ovarian cyst formation and disturbance of follicular development. Compared to control, animals in TiO_2_NP group have shown significant reduction of pregnancy rates and number of giving birth (p=0.04). TiO_2_NP caused significant reduction in oocyte number, fertilization rate, and pre-implantation embryo development (p<0.001). Furthermore, malondyaldehyde and estrogen hormone levels were significantly (p<0.01) increased in mice received TiO_2_NP.

**Conclusion:**

Our findings suggest that TiO_2_NP exposure induces alterations on mice ovary resulting in a decrease in the rate of embryo development and fertility.

## Introduction

Nanoparticles (NPs) are materials which at least one of their three dimensions is in the range of 1-100 nm and have unique physiochemical properties and functions which have obtained well interest in many areas in applications such as, food, industry, medical sciences, agriculture and military fields (1). Nanoparticles are being increasingly used and worries on their safety have been increased in scientific society and the general public, especially oral-use food related NPs (2, 3). NPs compared with same large chemical composites have low size and great surface to volume ratio which promote entrance to cells and also increase catalytic and biological activities (4). 

Titanium dioxide nanoparticle (TiO_2_NP) or titana is one of the most common materials used in various products and can be found in three forms, rutile, anatase and brookite (5, 6). Among them, anatase crystal is the most effective form. TiO_2_NP is used in products such as, paints, plastic, papers, cosmetics, clothings, electronics, toothpastes, and especially in sunscreens which due to their ability to block ultraviolet light protect the skin (7, 8). It also extensively used in food industry as a food additive. Thus, oral intaking is a major route from food products (9, 10), as a 75 kg adult human receive 15-37.5 mg/kg/day from food (7).

It has been proven that TiO_2_NP can be toxic to human and animal. Oral administration of this nanoparticle to the mice increases inflammation and disrupts the function of the liver, kidney and reproductive system (11-13) and also increases plasma glucose level (14, 15). Previous studies also have indicated that TiO_2_NP administration leads to aggregation in the vital organs such as, liver, brain, lung, spleen, and kidney (11, 16). Long term TiO_2_NP administration (90 days) to mice can be accumulated within ovarian cells, resulting in ovarian dysfunction, mating and pregnancy rate reduction, ovarian inflammation and follicular atresia (13, 17).

It also has been shown that TiO_2_NP was transferred to the brain of mice which can affect hormonal release from pituitary gland and female reproduction that leads to imbalance of sex hormone (11, 13). In addition, recent studies have shown that this nanoparticle is able to decrease sperm counts and motility and increases the number of abnormal sperms in epididymis (18, 19). Other studies indicated that TiO_2_NP inhibits follicular growth and oocyte maturation of rat (20), and also induces genotoxicity in Chinese hamster ovary cells in vitro (21). Hong and colleague demonstrated that oral administration of 100 mg/kg TiO_2_NP to pregnant mice can cross the blood fetal barrier and placental barrier and suppresses embryonic development and also induces fetal skeletal malformation (22). 

A considerable body of evidences in laboratory animal models have shown that TiO_2_NP administration can cause toxicity in the male reproductive system and induced impairment in testicular morphology and function but, to the best of our knowledge, there is no detailed study on toxic effects of subchronic oral administration of TiO_2_NP in the female reproductive system function, particularly in vitro fertilization (IVF). A few previous studies (13, 17) investigated the long term exposure (90 days) of TiO_2_NP on female reproductive system and IVF potential has not been evaluated. However, the effects of TiO_2_NP-induced in vitro fertility impairment have yet to be studied.

Thus, the current study was conducted to evaluate the adverse effects of oral administration of female mice to TiO_2_NP on the histological alterations of ovary, estrogen (E_2_) hormone levels, malondyaldehyde (MDA) concentration, pregnancy and IVF rates.

## Materials and methods

**Animals and treatments**

**Figure 1:**
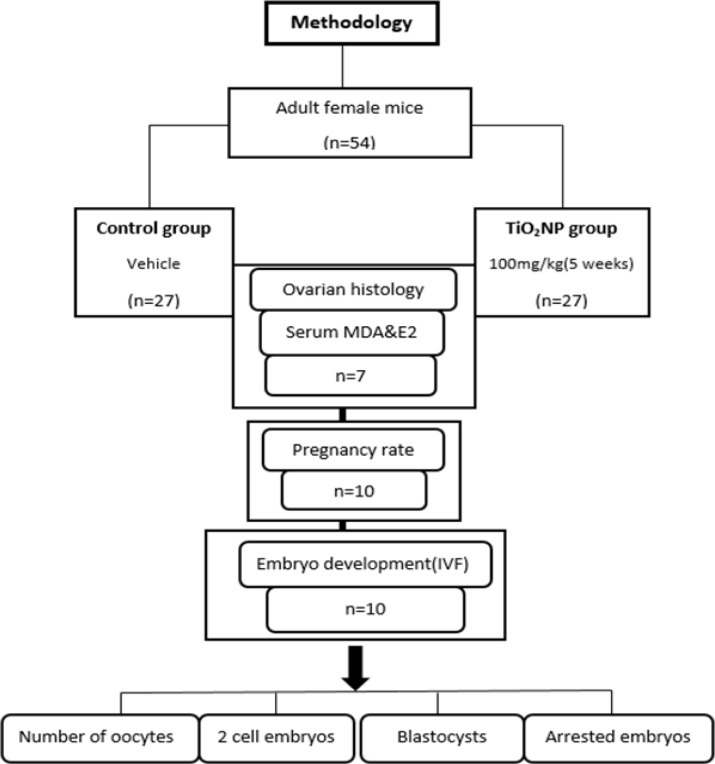
Flowchart for the experiment design.

In this experimental study, 54 adult female mice 10 wk (25±2 gr) were used. In order to evaluate IVF assay, sperms obtained from a normal male NMRI mouse was used. The mice were obtained from the Animal Center of Medical School. Animals were kept in stainless steel cages in a ventilated animal house. Room temperature of the animal house was maintained at 24±2^o^C with a 12 hr light/dark cycle. Food and water were given ad libitum. Prior the study, the mice were adapted to this environment for one wk. The mice were randomly categorized into 2 groups (n=27/each): control group received vehicle and test group was supplemented with TiO_2_NP solution orally at a dose of 100 mg/kg of body weight for 5 wk (Figure 1). 

**Preparation of TiO**_2_ **NP solution**

The size of the TiO_2_NP (99% anatase) was 10-25 nm and obtained from US Research Nanomaterials, Inc. It was prepared every wk in phosphate buffered saline (pH= 7.4) with 0.5% Tween 80 and immediately before using was dispersed and sonicated for 10 min. 

**Mating of animals**

We used 10 mice from each group to evaluate the effect of TiO_2_NP on the fertility and pregnancy potential rate. 24 hr after last day of administration, 3 control or test female mice were put in a common cage with 3 males for 11 days and percentage of pregnancy and numbers of newborns were evaluated (23).

**Histological assessments of ovary**

All histological assessments were done using standard laboratory methods. After 5 wk, mice were sacrificed and their ovaries were collected. The left ovary from each mouse was fixed with 10% formaldehyde and embedded in paraffin blocks and then sectioned at 5 µm thickness. The stained sections with hematoxylin-eosin (H&E) (Merck, Germany) were evaluated by at least two investigators unaware of the treatments under a light microscope. Seven mice per group used for this part of the study. 

**Serum E**_2_ **and MDA levels**

At the end of 5 wk, the blood serum of the animals used for histopathological examination were obtained for evaluating the levels of MDA (Zell Bio, GmbH, Germany) and E_2_ hormone (Monobind Inc., Lake Forest, CA, USA) using commercial ELISA kits according to the manufacturer`s protocols. 

**IVF assays**

We used 10 mice from each group for IVF assay. To collect mature oocytes from oviducts, each mouse was intraperitonealy injected with 10 IU pregnant mare`s serum gonadotropin (PMSG, Folligon, Netherland) and 10 IU human chorionic gonadotropin (hCG, Folligon, Netherland) after 48 hr. The mice were sacrificed 13-14 hr after human chorionic gonadotropin injection, and then the cumulus-oocyte complexes were obtained from both ampulla of oviducts and transferred to a petri dish containing human tubular fluid (HTF) medium with 4 mg/ml bovine serum albumin (BSA), (Sigma, St. Louis, USA). 

The epididymal sperm were obtained from the caudal epididymis of a male adult mouse. Sperm suspensions were placed in HTF-BSA medium and capacitated by incubation at 37^o^C and 5% CO_2_ for at least 1 hr. Then 1×10^6^ sperms/ml were added to 500 µL fertilization droplets of HTF-BSA medium containing oocytes. Mineral oil was used to cover droplets. Under inverted microscope, fertilized oocytes were assessed by presence of male and female pronuclei and polar body. After 24 hr of zygotes culture, the percentage of two-cell embryo was assessed and embryonic growth was evaluated after 120 hr (24). In this study the rate of the arrested embryos was categorized into three types: 

Type I: embryos with full fragmented balstomeres. 

Type II: embryos with partially lysed or fragmented blastomeres. 

Type III: embryos with some fragmented blastomeres.

**Ethical consideration**

The study was approved and performed according to Ethical Committee Guidance for Research at Laboratory Animals of Urmia University of Medical Sciences (1394-0-32-1791).

**Statistical analysis**

The results of IVF were analyzed by 2 proportional test using Minitab software version 15.1 (Minitab Inc., PA, USA). Other results were examined by independent Student`s t-test using SPSS software (Statistical Package for the Social Sciences, version 16.0, SPSS Inc, Chicago, Illinois, USA). All results were shown as means±standard deviation (SD) and a p<0.05 was determined as statistically significant.

## Results

**Pregnancy rate and number of newborns**

As shown in table I, pregnancy rate and number of newborns were significantly decreased in TiO_2_NP exposed group compared with control group (p=0.04).

**MDA concentration**

To determine whether orally administrated TiO_2_NP affect lipid peroxidation in mice, serum MDA levels were measured. As shown in table I, the level of MDA in TiO_2_NP group was significantly (p<0.01) higher than control group.

**E**_2_ **hormone levels**

The levels of E_2_ hormone in the blood serum of female mice is presented in table I. Orally TiO_2_NP exposure caused significant increase in E_2_ levels compared to control (p<0.01).

**Histological observations**

Histological examination of the ovaries in the control group showed normal architecture of ovarian tissue with normal follicles at different stages of development. Several pathological changes were observed in the TiO_2_NP group, including degeneration and reduction of ovarian follicules, ovarian cyst formation, and follicular development impairment suggesting that ovaries have been damaged by TiO_2_NP exposure.

**Fertilization rate and embryonic development**

Data of oocytes number, IVF rate, and pre-implementation embryonic development in studying groups are presented in table II. The results indicated that number of oocytes, percentages of fertilization, two-cell embryos (as an indicator of cleavage initiation), and embryos in blastocyst stage in the TiO_2_NP group were significantly lower than control group (p<0.001). Compared with the control group, in TiO_2_NP group the total percentages of arrested embryos were significantly higher (p<0.001).

**Table I:** The effects of TiO_2_NP on pregnancy rate, number of newborns, E2 and MDA levels of mice after oral administration with 100 mg/kg body weight for 5 wk

**Index**	**Control**	**TiO**_2_ **NP (100mg/kg)**
Pregnancy rate (%)	100	70
Number of newborns	8.9 ± 1.19	7.2 ± 0.92 a
MDA (nmol/ml)	1.43 ± 0.07	1.8 ± 0.24^b^
E_2_ (pmol/l)	61.56 ± 5.71	73.74 ± 7.56^b^

Data of pregnancy rate presented as% and the rest presented as means ± SD.

independent-samples t-test (n=10)

TiO_2_NP: Titanium dioxide nanoparticle

MDA: Malondyaldehyde

E_2_: Estrogen

a Significantly different from the control (p<0.05)

b Significantly different from the control (p<0.01)

**Table Π:** The number of oocytes and percentages of fertilized oocytes and embryos (two- cell and blastocyst) of mice after oral exposure with 100 mg/kg TiO_2_NP for 5 wk

Groups	Oocytes	Fertilized ooocytes	Two cells	Blastocysts	Arrest	Arrest type I	Arrest type II	Arrest type III
**Control**	**271** a	**258** **(95.2)** b	**234** **(90.7)** a	**155** **(60.08)**^a^	**103** **(39.9) **a	**3** **(0.78) **a	**7** **(2.71) **a	**94** **(36.43) **b
**TiO2 NPS**	**194**	**173** **(89.17)**	**102** **(52.95)**	**37** **(21.39)**	**136** **(78.6)**	**34** **(19.65)**	**26** **(15.03)**	**76** **(43.93)**

Data of oocytes presented as n and the rest presented as n (%)

2 proportional test (n=10)

TiO_2_NP: Titanium dioxide nanoparticle

a Significantly different from the control (p<0.001)

b Significantly different from the control (p<0.05)

**Figure 2:**
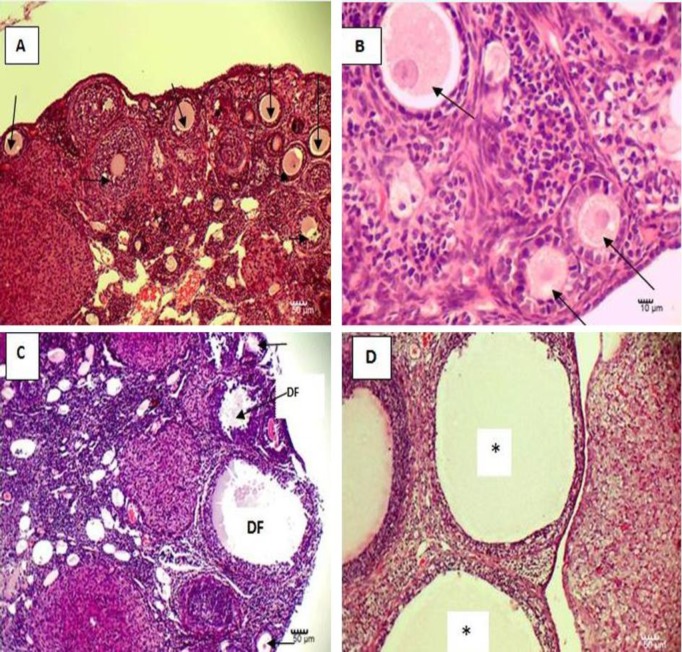
Effects of oral administration of TiO_2_NP on mice ovary: A and B, control group showing normal structure with normal follicles; C and D, ovarian tissue in TiO_2_NP exposed group showing reduction and degenerating of follicles and cyst formation. Arrows indicate follicle, DF; degenerating follicle, *; cyst in ovary. (A and D ×100; B and C ×400 magnification).

## Discussion

The results of the present study showed that oral administration of TiO_2_NP with 100 mg/kg for 5 wk caused reproductive system toxicity. The findings indicated that TiO_2_NP administration led to follicolugenesis impairment and pregnancy and in vitro fertilization rates reduction.

It has been proven that orally administration with TiO_2_NP accumulated in mouse ovary and lead to ovarian dysfunction such as atresia of primary and secondary follicles and also ovarian apoptosis induction (17). In this study, adult female mice were treated for 5 wk. Considering the time of the mouse estrous cycle is 4-5 days, in this study we exposed the mice for about 5 cycles to investigate the toxic effects of TiO_2_NP.

Our findings indicated that subchronic TiO_2_NP exposure resulted in an increase in the serum E_2_ concentration that was agreed with previous studies which also showed significant reduction of levels of luteinizing hormone (LH), testosterone, progesterone, and follicle-stimulating hormone (FSH) (13, 17). Decreased pregnancy rate in the present study may be related to imbalance of sex hormone concentrations. It is well known that there is a direct relationship between normal fertility and ovarian follicular development with the levels of sex hormones (17). In females, progesterone plays a key role in ovulation, implantation, and pregnancy (25) and also has been proven to be important for ovulation through increasing of proteolytic enzymes production (26). Furthermore, LH and FSH are the most important hormones of hypothalamic-pituitary-gonadal axis, which regulates the production of gametes and fertility. E_2_ hormone also facilitates induction of receptor systems for FSH and LH in the granulosa cells (27). Thus, reduction of folliculogenesis and fertility by TiO_2_NP administration may be due to impairments of sex hormones release such as E_2_, FSH, LH, and progesterone (17). Increased E_2_ levels in the current study and in previous studies may be associated with the activation of cytochrome p450 aromatase which is responsible for converting testosterone to E_2_, but proving this needs further investigation (28).

Our findings also indicated that the in vitro fertility potential and embryonic development in mice administrated with TiO_2_NP were lower than those of the control mice and also the percentage of arrested embryos was significantly higher after TiO_2_NP administration. It is clear that embryonic development in both in vitro and in vivo is associated to quality of both gametes (oocyte and sperm) (29). The previous studies indicated that chronic oral exposure of TiO_2_NP for 90 consecutive days led to significant accumulation in the mouse ovaries and altered the ovarian stromal ultrastructure such as, mitochondrial alterations including swelling and crista break, nucleus chromatin condensing, and nuclear membrane deformity. These alterations suggest that TiO_2_NP administration caused ovarian stromal cells apoptosis (17). Extracellular matrix of ovary and also the stromal cells play an important role in tissue integrity, normal function of the ovary, and follicular development. Therefore, the reduction in mice fertility potential following TiO_2_NP administration may be due to disturbances in the organization of the ovarian cells ultrastructure and also hormonal imbalance. 

In the current study, we observed histological alterations in ovarian tissue caused by TiO_2_NP administration including impairment in folliculogenesis and degenerating of follicles resulted in decrease of mature oocytes. These findings were confirmed by IVF assay results which indicated that the number of oocytes collected from these mice were significantly lower than those of the unexposed mice. Moreover, the quality of these retrieved oocytes were lower in treatment group. Thus, decreased the number of two cell embryos and mating capacity of female mice following TiO_2_NP administration were closely associated with these findings. To our knowledge, no published data was available about the toxic effects of TiO_2_NP on in vitro development of female mice embryos. Thus, we are not able to compare the findings obtained from the present study with other studies.

In this study, the TiO_2_NP exposed mice showed a statistically significant increase in MDA levels. Previous studies showed that TiO_2_NP increase reactive oxidative species (ROS) levels in mice (30, 31). MDA produces following reaction between ROS and lipids, and its level is increased after ROS elevation (32). Thus, MDA is commonly used as ROS-related marker (30). In the present study, the analysis of serum MDA levels after 5 wk showed that its level in the TiO_2_NP supplemented group was higher than control group which was consistent with previous report (30).

The toxic effects of TiO_2_NP exposure in vivo model may be caused through both direct and indirect pathways (33). In direct pathway, TiO_2_NP is able to enter the blood circulation after oral exposure and then be distributed to different tissues such as liver, spleen, kidney, lung (11) and also ovary (13). Thus, in current study, it seems that the accumulation of this nanoparticle in ovaries caused toxicity which was demonstrated by biochemical and histological alterations. 

In indirect pathway, oral administration of TiO_2_NP affects ovary through increasing ROS levels and also through triggering inflammatory responses. According to previous studies, increased levels of inflammatory cytokines including tumor necrosis factor alpha, interleukin-6, and interleukin-8 in the blood serum of mice and rats after oral administration of TiO_2_NP suggests significant inflammatory responses (30, 34). As mentioned earlier, we found a significant elevation in the level of MDA which was consistent with previous studies. Thus, stress oxidative induced by TiO_2_NP exposure probably is responsible for female reproductive system damage.

Our study is not without limitation. We did not measure levels of other sex hormones (LH and FSH) and, we used only one dose of TiO_2_NP for 5 wk. Therefore, we propose to apply lower doses of exposure in future studies.

## Conclusion

The results of the current study indicate that subchronic oral administration of TiO_2_NP may affect pregnancy rate, in vitro fertility and embryo development and also induces ovarian tissue damage and ultimately leads to mice ovarian dysfunction which may be associated with lipid peroxidation and imbalance of sex hormones. Therefore, these data can provide useful information on the risk of application of TiO_2_NP in young females, especially during childbearing years.
